# Supplementary material for: At-home blood collection and stabilization in high temperature climates using homeRNA
Source: Front Digit Health. 2022 Aug 9;4:903153. doi: 10.3389/fdgth.2022.903153 (PMC9405416; doi:10.3389/fdgth.2022.903153)

Supplementary Material

# Supplementary Figures and Tables

**1.1 Supplementary Tables**

**Table S1: Participant and sampling information for the Western and South Central USA pilot study**

| **Time (h)** | **Event / Procedure** | **External Temperature (℃)** | **Location** |
| --- | --- | --- | --- |
| 0 | Overnight hold at room temperature | 21 | Lab, Research |
| 27 | Start in lab | 21 | Lab, Research |
| 27.25 | Transport outside to car | 26 | Outside |
| 27.3 | In car - A/C started | 35.5 | Indoor parking |
| 27.4 | In car - A/C on | 27 | Indoor parking |
| 27.5 | In car - driving with some sun exposure | 33 | Driving |
| 28 | In car - A/C stopped | 31 | Outdoor parking - moderate sun exposure |
| 28.1 | In car - A/C restarted | 37 | Outdoor parking - moderate sun exposure |
| 28.25 | Car stopped - A/C off | 30 | Outdoor parking - covered |
| 29 | Car restarted - A/C on | 41 | Outdoor parking - covered |
| 29.19 | Car stopped - A/C off | 32 | Home parking - covered |
| 29.42 | Car restarted - A/C on | 40 | Home parking - covered |
| 29.49 | Car stopped - A/C off - Walking to Lab | 32 | Indoor parking |
| 29.54 | Enter building | 36 | Research |
| 29.56 | Office | 32 | Office, Research |
| 30 | Transferring blood from stabilizer tube to store | 21 | Lab, Research |
| 30.3 | Storing sample | -80 | Lab, Research |

**1.2 Supplementary Figures**


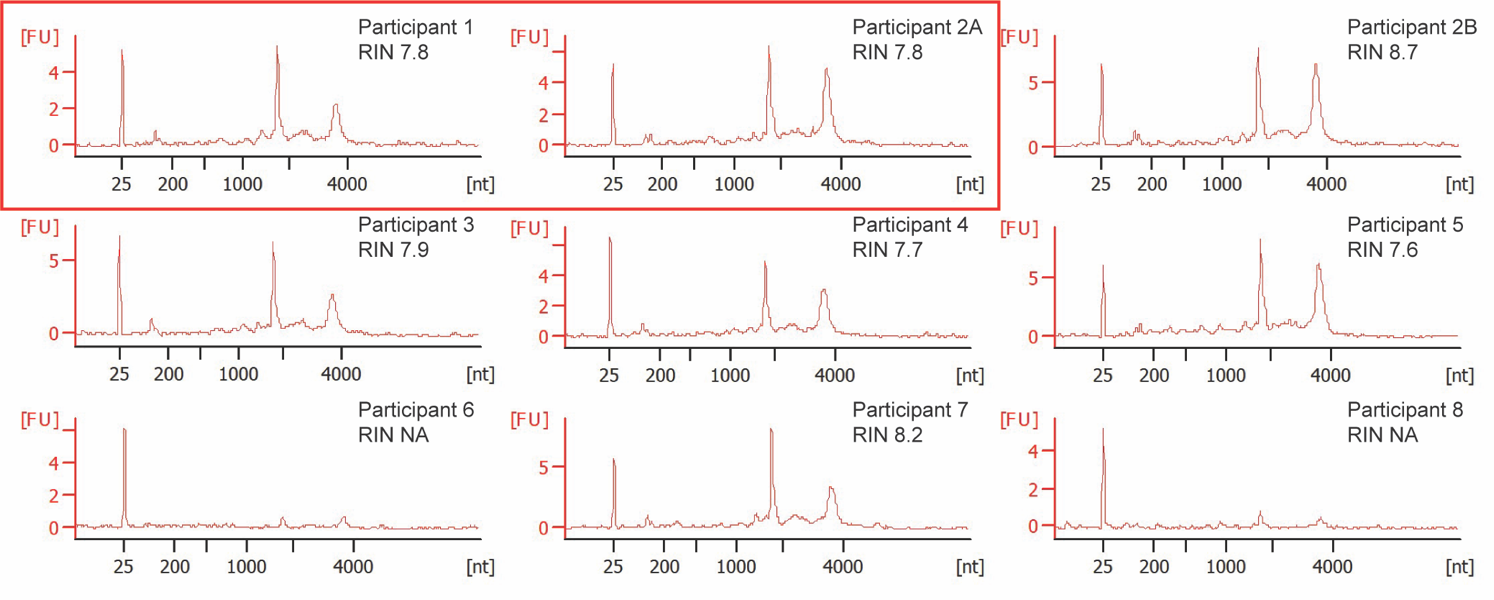


**Figure S1. Electropherograms of isolated RNA from stabilized *home*RNA samples in Doha, Qatar.** Electropherograms were obtained as raw data using a RNA 6000 Nano Kit on an Agilent 2100 bioanalyzer, which uses fluorescence to detect the marker (~25 nt), 18S rRNA fragments (~1900 nt), and 28S rRNA fragments (~3900 nt). The RIN algorithm then uses these peaks to assign a RIN value to the corresponding sample. The electropherograms are used to generate a gel image, as seen in **Figure 2B**. Samples 1 and 2A in the red box correspond to control samples.

**
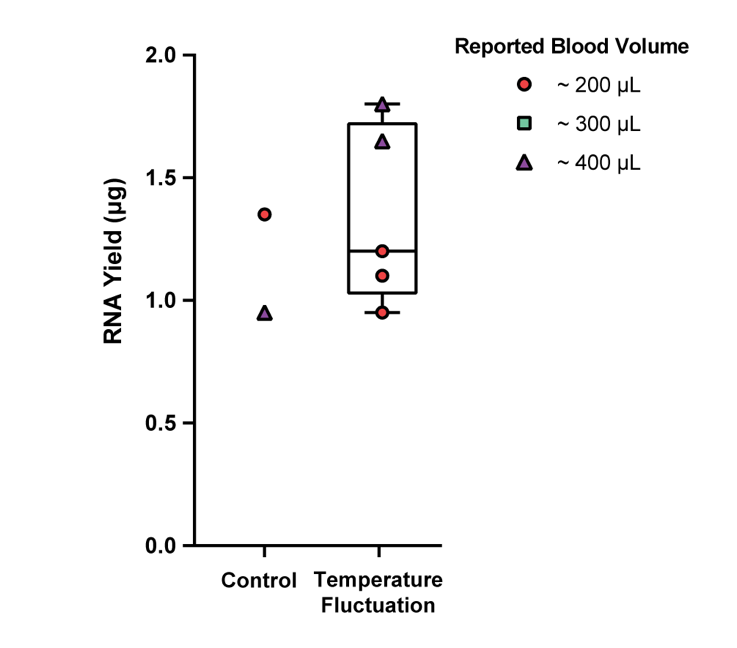
**

**Figure S2. Yield of isolated RNA from stabilized *home*RNA samples in Doha, Qatar.** Reported yields were obtained from bioanalyzer measurements by taking the reported concentration from the first 50 μL elution. For the two samples (from participant 6 and 8) that did not have scorable RIN values, yields were not included on the graph since their concentrations were below the lower limit of the qualitative range of the RNA 6000 Nano kit (5 ng/μL). However, for reference, the bioanalyzer reported 4 ng/μL for participants 6 and 8. While concentrations from the Agilent 2100 Bioanalyzer are not typically used for obtaining yield, a threshold value of 200-500 ng total yield is typically needed for downstream analysis such as RNA sequencing. Therefore, an exact value is less important and the bioanalyzer can be used to obtain approximate RNA concentration ranges.


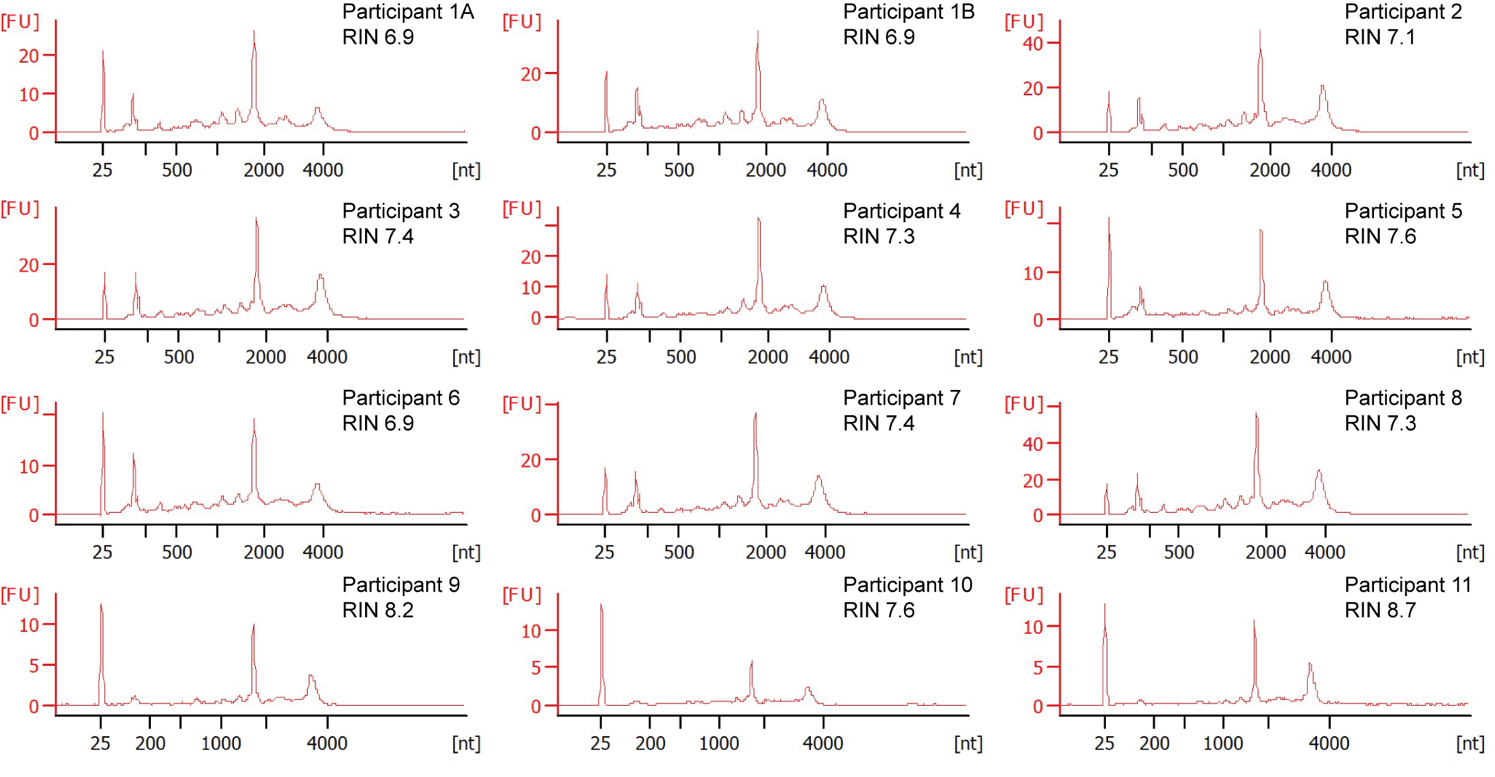


**Figure S3. Electropherograms of isolated RNA from stabilized *home*RNA samples in the Western and South Central USA.** Electropherograms were obtained as raw data using a RNA 6000 Pico Kit for samples 1-8 and a RNA 6000 Nano Kit for samples 9-11 on an Agilent 2100 bioanalyzer. For samples 1-8, the RNA 6000 Pico kit uses fluorescence to detect the marker (~25 nt), 5S rRNA fragments (~150 nt), 18S rRNA fragments (~1900 nt), and 28S rRNA fragments (~3900 nt). The RNA 6000 Nano kit detects the same peaks with the exception of the 5S rRNA fragment peaks. The RIN algorithm then uses these peaks to assign a RIN value to the corresponding sample. The electropherograms are used to generate a gel image, as seen in **Figure 3A**.


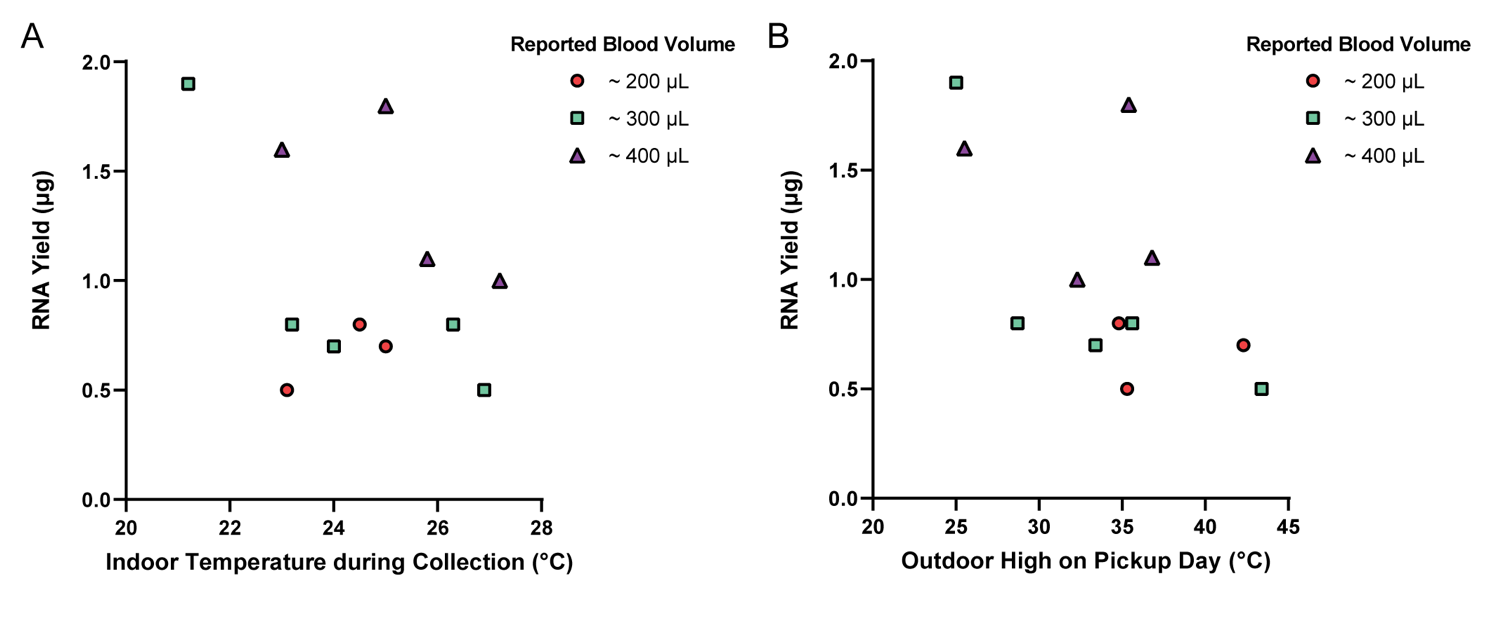


**Figure S4. Total yield of isolated RNA from stabilized *home*RNA samples collected, stabilized, and shipped at high temperatures in Western and South Central USA.** Reported total yields were obtained by measuring both 50 μL elutions from the RNA isolation protocol (see Methods and Materials) on Cytation 5 Multi-Mode Reader and adding them together to obtain a total yield. Each total yield is reported with the corresponding A) indoor ambient temperature at the time of collection and B) reported outdoor temperature high on the pickup day.


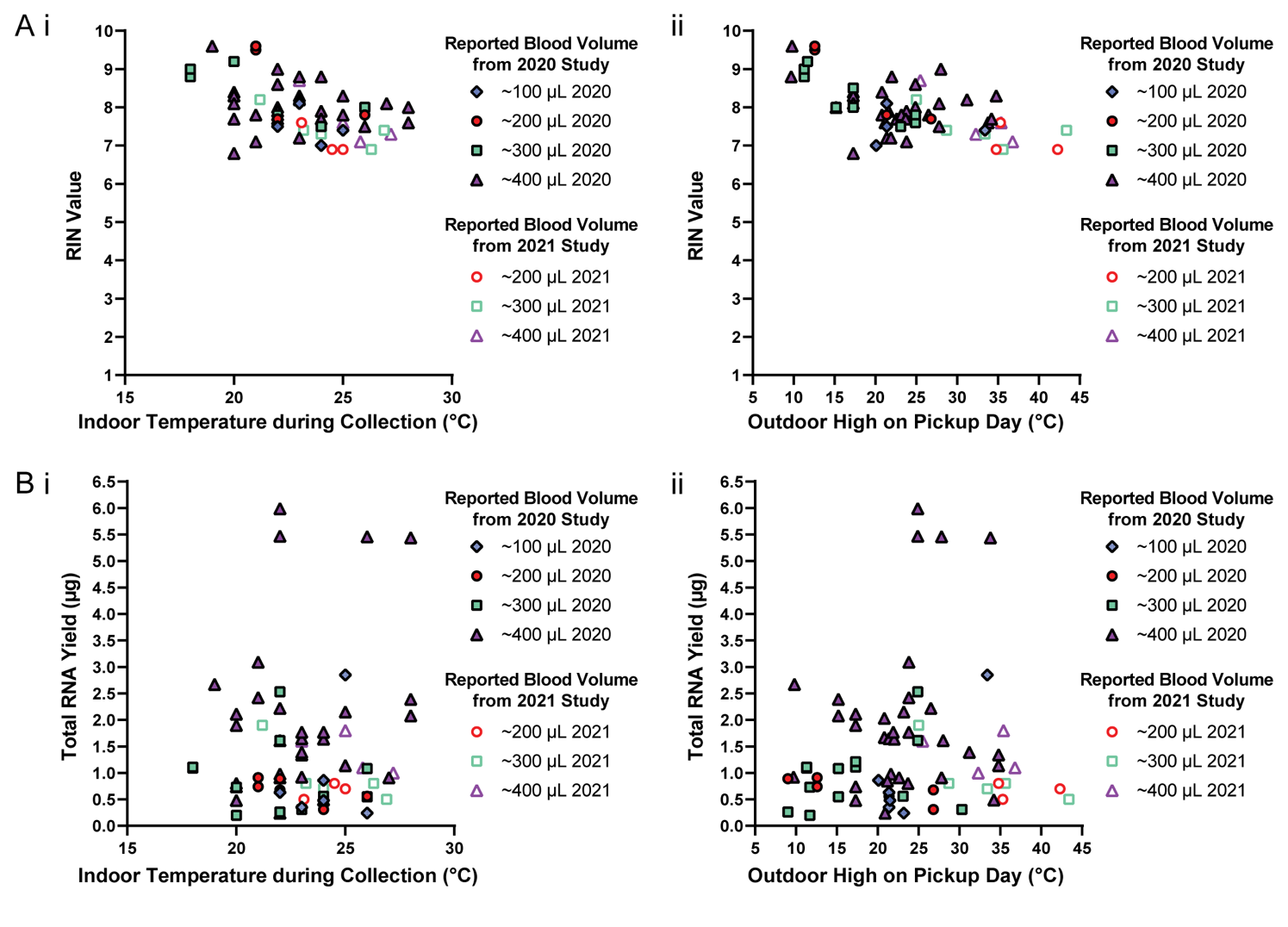


**Figure S5. RNA quality and quantity versus temperature, combining data from the present study (summer 2021) from our previous pilot study (summer 2020, Haack, Lim *et al.* 2021)** (1). A) RIN values versus i) indoor temperature and ii) outdoor high on pickup day. A) Total RNA yield versus i) indoor temperature and ii) outdoor high on pickup day.


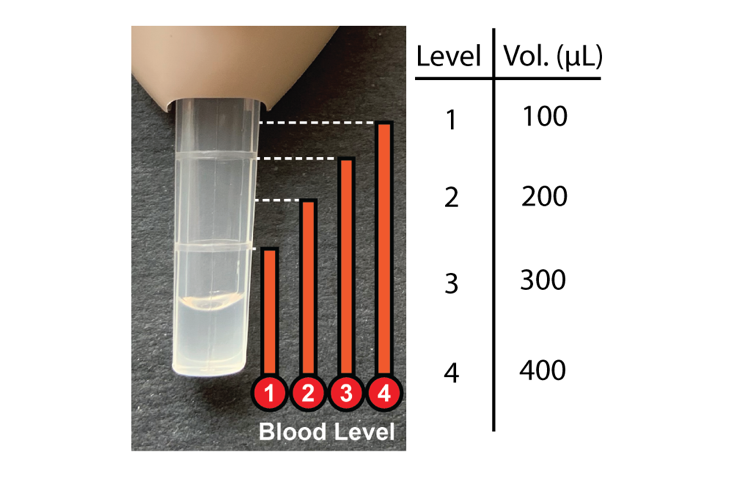


**Figure S6. Reported blood level volumes.** Study participants for both the Qatar and Western and South Central USA groups were asked to report the approximate volume with each blood collection with the Tasso-SST™ based on this picture provided in the survey that they completed with sampling. Image was reprinted with permission from Haack, Lim *et al.* *home*RNA: A Self-Sampling Kit for the Collection of Peripheral Blood and Stabilization of RNA**.** *Anal. Chem.* 2021, 93, 39, 13196–13203. Copyright 2021 American Chemical Society. (1)

# Appendix 1: *home*RNA kit components and instructions (all materials reprinted from Haack, Lim *et al.* SI with permission from Haack, Lim *et al.* *home*RNA: A Self-Sampling Kit for the Collection of Peripheral Blood and Stabilization of RNA. *Anal. Chem.* 2021, 93, 39, 13196–13203. Copyright 2021 American Chemical Society. for ease of reference) (1).


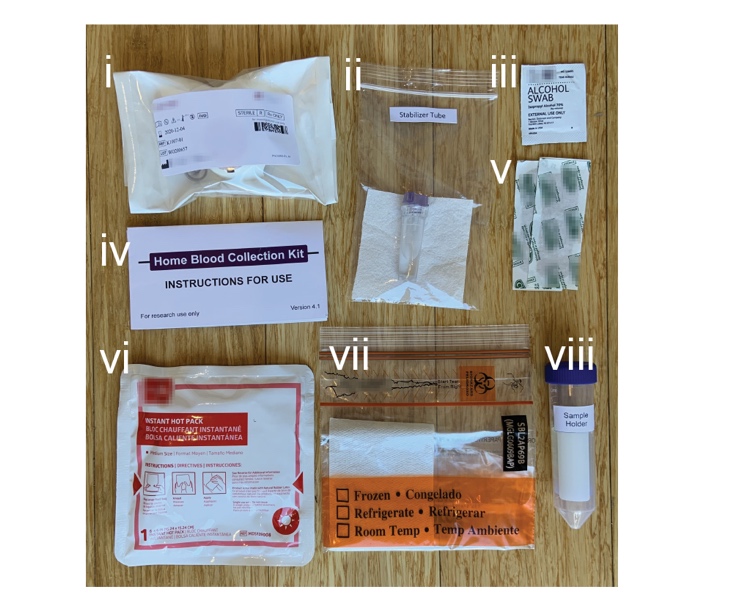


**Figure S1. Components of the *home*RNA kit.** Kit components include i) the Tasso-SST™ device, ii) the stabilizer tube containing RNA*later*™ iii) alcohol wipes iv) instructions for use, v) sterile bandages, vi) hot pack for warming the arm prior to application of the Tasso-SST™, vii) sample return bag and viii) sample holder with 3D printed insert to hold the sample tube in place.

**Table S1. Components of the *home*RNA blood kit.**

| **Kit Component** | **Manufacturer(s)** | **Quantity** |
| --- | --- | --- |
| Sterile Tasso-SST™ blood collection device | Tasso, Inc. | 1 |
| RNA stabilizer tube | Our Lab | 1 |
| Instant heat pack | Medline Industries, Inc. | 1 |
| Sterile Alcohol Wipe | Covidien, BD | 2 |
| Sterile bandage | Band-Aid, Curad | 1 |
| Specimen transport bag with absorbent pad | Minigrip | 1 |
| 50 mL conical tube with stabilizer tube insert | BD, Our Lab | 1 |
| Instructions for use | Our Lab | 1 |
| Blood-stabilizer mixing instruction card | Our Lab | 1 |


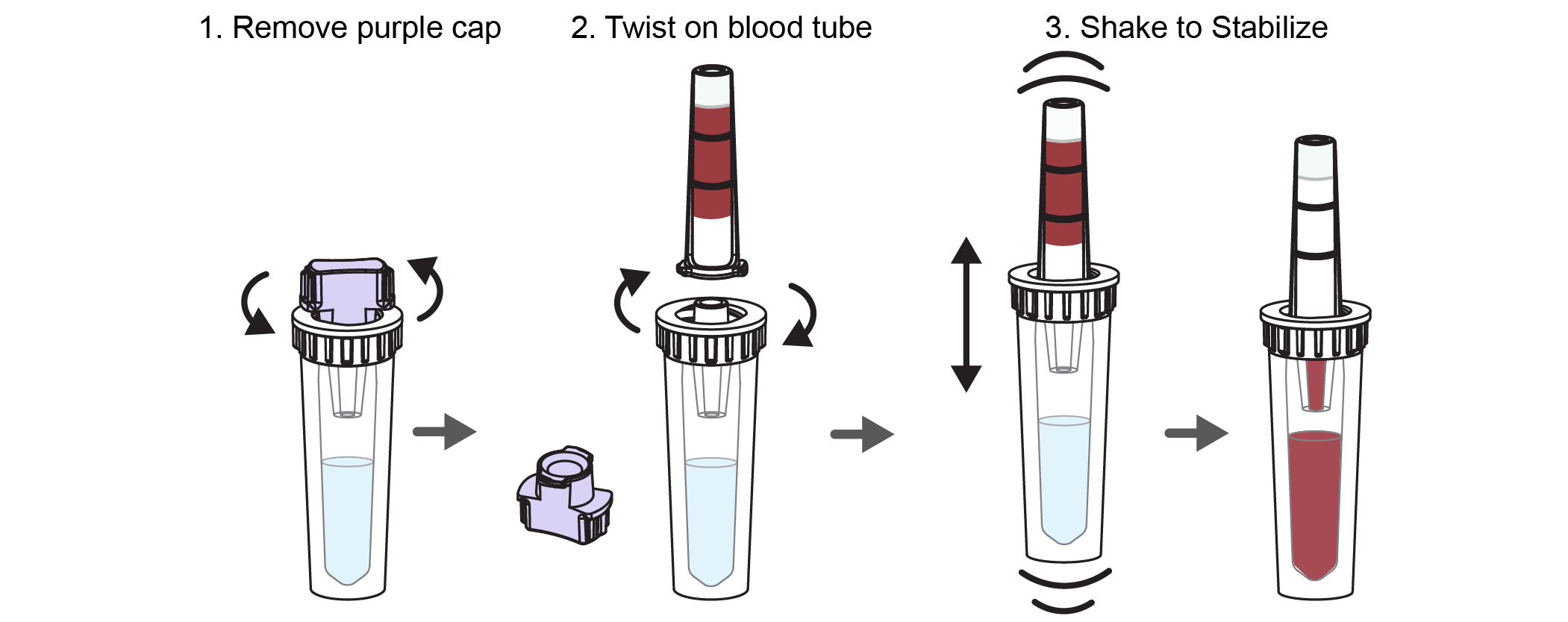


**Figure S2. Schematic workflow of the stabilization process.** The steps for stabilizing a blood sample collected with the Tasso-SST™ are as follows: 1) removal of the purple cap, 2) twisting on the Tasso-SST™ blood tube with the blood sample, 3) shaking up and down vigorously to mix the sample with the stabilizer resulting in RNA stabilized blood.

**Instructional video for *home*RNA**

An instructional video for using *home*RNA can be found at the following link: <https://youtu.be/iV3GZ8SmmuM>

**Instructions for Use (IFU):**

The IFU for the self-collection and stabilization of blood using *home*RNA is shown below (Pg. 10 – 11). Figure was reprinted with permission and adapted from Haack, Lim *et al.* *home*RNA: A Self-Sampling Kit for the Collection of Peripheral Blood and Stabilization of RNA**.** *Anal. Chem.* 2021, 93, 39, 13196–13203. Copyright 2021 American Chemical Society. (1)

# References

Haack AJ, Lim FY, Kennedy DS, Day JH, Adams KN, Lee JJ, Berthier E, Theberge AB. homeRNA: A Self-Sampling Kit for the Collection of Peripheral Blood and Stabilization of RNA. *Anal Chem* (2021) **93**:13196–13203. doi: 10.1021/acs.analchem.1c02008


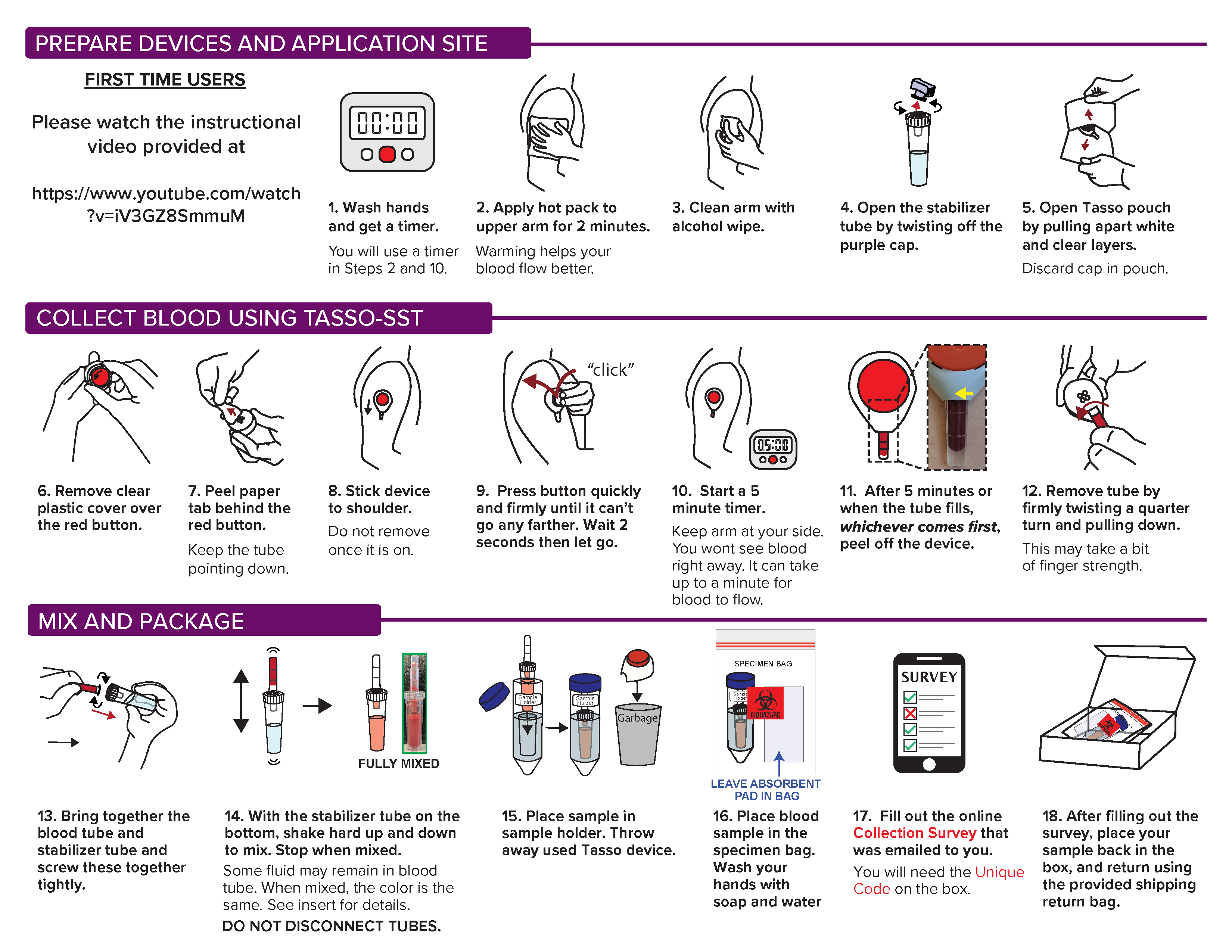


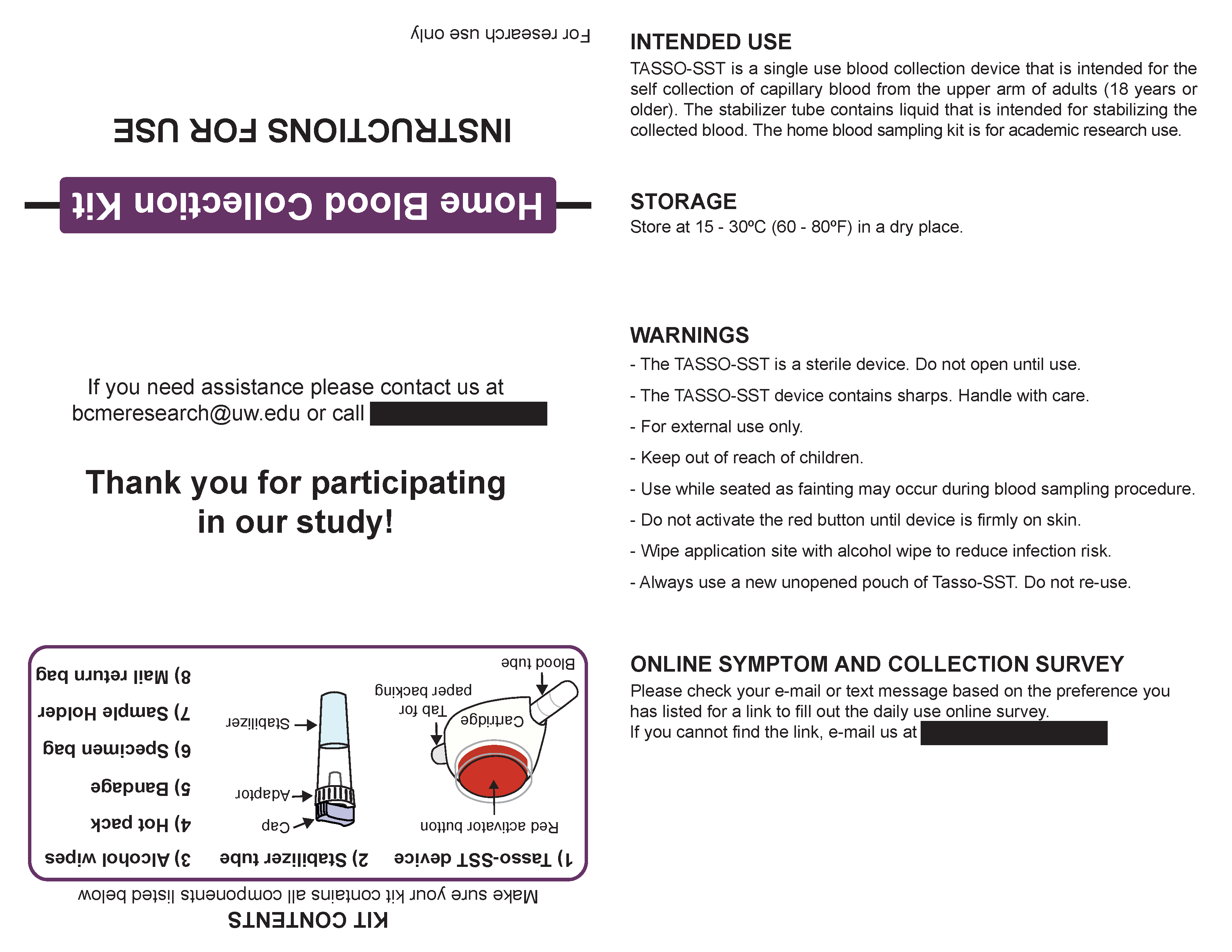

Supplement: Supplementary file 1 [file Table_1_v1.docx]
